# A novel *LAMP2* initiation codon mutation causes Danon Disease: a case report

**DOI:** 10.3389/fcvm.2025.1699732

**Published:** 2025-10-08

**Authors:** Qingchuan Li, Zhihong Sun, Zeping Qiu, Yanjia Chen, Wei Jin

**Affiliations:** ^1^ Department of Cardiovascular Medicine, Ruijin Hospital, Shanghai Jiao Tong University School of Medicine, Shanghai, China; ^2^ Heart Failure Center, Ruijin Hospital Lu Wan Branch, Shanghai Jiao Tong University School of Medicine, Shanghai, China

**Keywords:** Danon Disease, *LAMP2* gene, initiation codon mutation, false-positive scintigraphy, genetic test, gender differences, case report

## Abstract

Danon Disease (DD) is a rare X-linked inherited disorder caused by severe deficiency of lysosome-associated membrane protein-2 (LAMP-2), encoded by the *LAMP2* gene. Characteristic clinical features include a triad of cardiomyopathy, skeletal myopathy and cognitive impairment in males. Females usually exhibit milder, cardiac-predominant manifestations later in life. In this case, we report a 30-year-old woman with a novel suspected pathogenic *LAMP2* mutation (c.1A > T, initiation codon mutation). She developed Wolff-Parkinson-White (WPW) syndrome in her twenties, acute heart failure post cesarean section at age 29, and persistent left ventricular hypertrophy. Positive result of Technetium-99 m pyrophosphate (^99m^Tc-PYP) scintigraphy strongly indicated transthyretin amyloid cardiomyopathy (ATTR-CM). However, whole-exome sequencing (WES) identified the novel A to T transition in initiation codon (c.1A > T) of *LAMP2* gene, establishing the diagnosis of DD and revealing the false-positive result of PYP scintigraphy in DD.

## Introduction

Danon Disease is a rare X-linked dominant disorder characterized by severe cardiomyopathy, skeletal myopathy, and cognitive impairment, often accompanied by retinopathy and multi-system (neurologic, hepatic, gastrointestinal, pulmonary) involvement (1). The disease stems from deficiency of lysosome-associated membrane protein-2 (LAMP-2), encoded by the X-chromosomal *LAMP2* gene (Xq24). LAMP-2, essential for autophagosome maturation via fusion with lysosomes and endosomes, is predominantly localized to these organelles' membranes (2, 3). Deficiency of its cardiac/skeletal muscle-enriched isoform LAMP-2B impairs autophagy, causing autophagosome accumulation, cellular hypertrophy, and ultimately myocyte death with fibrosis (1, 2).

Due to its X-linked dominant inheritance, Danon Disease demonstrates significant gender differences in phenotypic expression (4). Male patients typically develop cardiac symptoms in their mid-twenties, often progressing to severe heart failure requiring transplantation; they also frequently exhibit the classic triad and retinopathy. In contrast, cardiomyopathy in female patients usually manifests during adulthood, typically as left ventricular hypertrophy and conduction abnormalities; extracardiac manifestations are often absent or mild (4).

Here, we describe a 30-year-old woman presenting with mild left ventricular hypertrophy and conduction abnormalities, Whole-exome sequencing (WES) identified a novel initiation codon mutation in the *LAMP2* gene, strongly suggesting the diagnosis.

## Case presentation

A 30-year-old woman was referred to our hospital for cardiac assessment. A summarized timeline of key clinical events is presented in Table 1.

**Table 1:** Timeline of the course of disease.

Time/Age	Manifestations/Events	Management	Therapy
Early 20s	Diagnosed with WPW Syndrome	Received a catheter ablation procedure	No pharmacotherapy
2019/02/20, aged 28	ECG showed no abnormality during pregnancy		
2019/08/23, aged 28	Fever, cough, acute respiratory and cardiac failure after cesarean section, ECG showed reduced LVEF of 48%, with 13mm-thick interventricular septum and 12mm-thick posterior wall	Intensive Care Unit admission	
2019/10/05, aged 28	Mildly improved LVEF of 52%, with 13mm-thick interventricular septum and 12mm-thick posterior wall		Metoprolol 12.5 mg twice daily, Perindopril 4 mg once daily
2019/11/20, aged 28	LVEF improved to 60%, with 12mm-thick interventricular septum and 12mm-thick posterior wall		As above
2020/04/02, aged 29	LVEF 59%, with 13mm-thick interventricular septum and 13mm-thick posterior wall		As above
2020/09/17, aged 29	LVEF 55%, with 13mm-thick interventricular septum and 13mm-thick posterior wall		

In her early twenties, she was diagnosed with Wolff-Parkinson-White (WPW) Syndrome and underwent catheter ablation procedure at a local hospital. Despite persistent intermittent palpitations, she received no pharmacotherapy. In 2019, at 39 weeks' gestation, she developed a fever requiring intensive care unit admission for acute respiratory and cardiac failure following emergency cesarean section. Echocardiography (ECG) showed mild left ventricular hypertrophy with thickness of 13 mm in interventricular septum and 12 mm in posterior wall, and impaired systolic function with left ventricular ejection fraction (LVEF) of 48%. Diagnosed with peripartum cardiomyopathy, she was initiated on Metoprolol 12.5 mg twice daily and Perindopril 4 mg once daily. Follow-up echocardiography revealed improved left ventricular systolic function (LVEF 60%), although left ventricular wall thickness remained unchanged.

The patient presented to our clinic seeking evaluation regarding her suitability for another pregnancy. During the consultation, we elicited a concerning family history. She reported three miscarriages and the premature death of her firstborn son at age 1 due to undetermined cardiac causes. Her mother had suspected hypertrophic cardiomyopathy and died at age 30. The family pedigree (Supplementary Figure S1) is suggestive of an X-linked inheritance pattern for cardiomyopathy.

**Figure 1:**
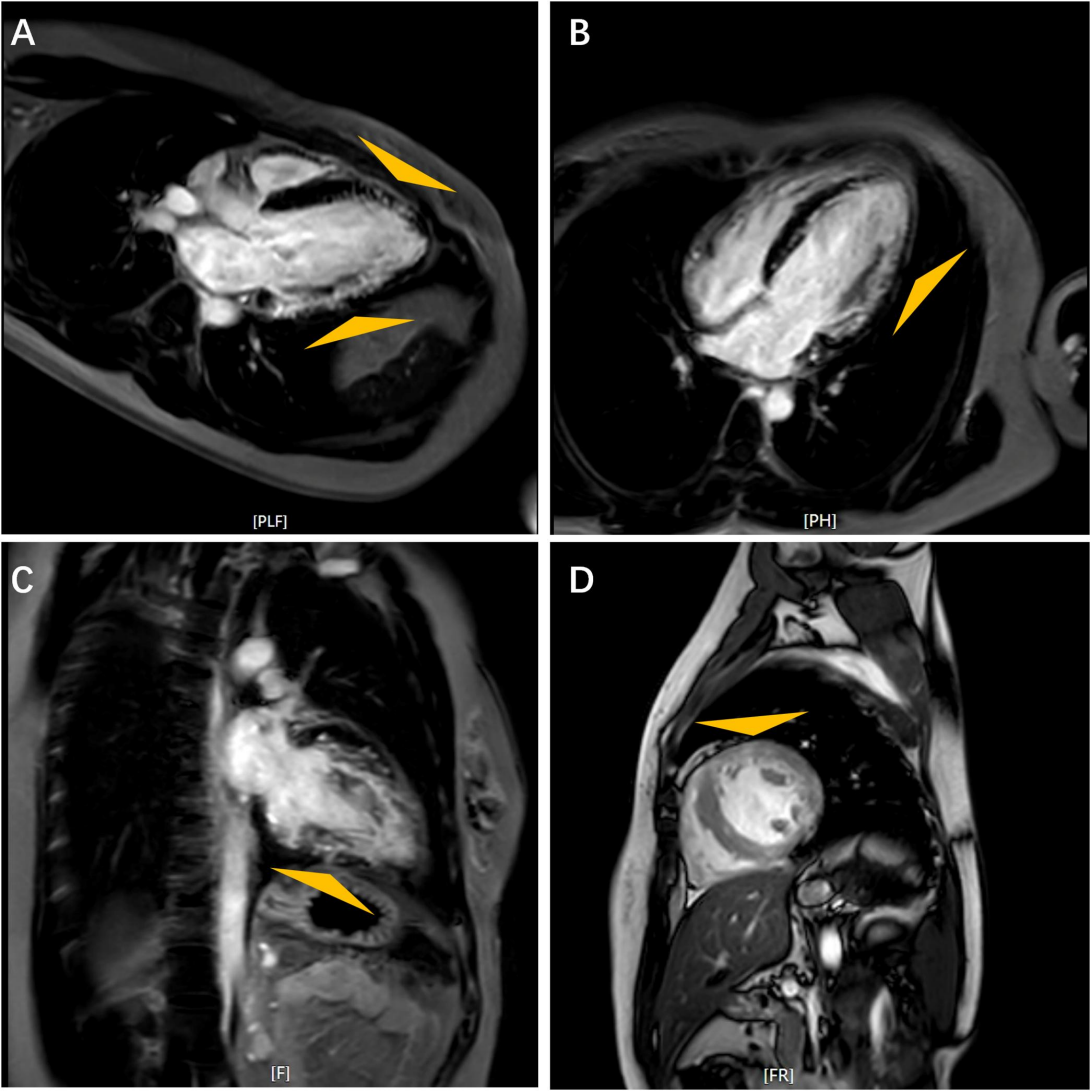
Cardiac magnetic resonance images. Feather-like delayed gadolinium enhancement in the left ventricular wall as indicated by triangles. Long Axis **(A)**; Horizontal Long Axis **(B)**; Four-Chamber View **(C)**; Short Axis **(D).**

On admission, the patient's vital signs were within normal range, and she demonstrated no clinical sign of heart failure. Laboratory findings are detailed in Supplementary Table S1. Electrocardiogram (EKG) revealed a short PR interval of 106 ms, ventricular pre-excitation and sinus rhythm with a heart rate of 67 beats per minute (Supplementary Figure S2). ECG indicated mild left ventricular hypertrophy with thickness of 12 mm in interventricular septum and posterior wall, and impaired systolic function with an estimated LVEF of approximately 48% (Supplementary Figure S3). Further evaluation with cardiac magnetic resonance (CMR) imaging revealed diminished left ventricular wall contraction and late gadolinium enhancement (LGE) involving the subendocardium (indicated by triangles in Figure 1).

**Figure 2:**
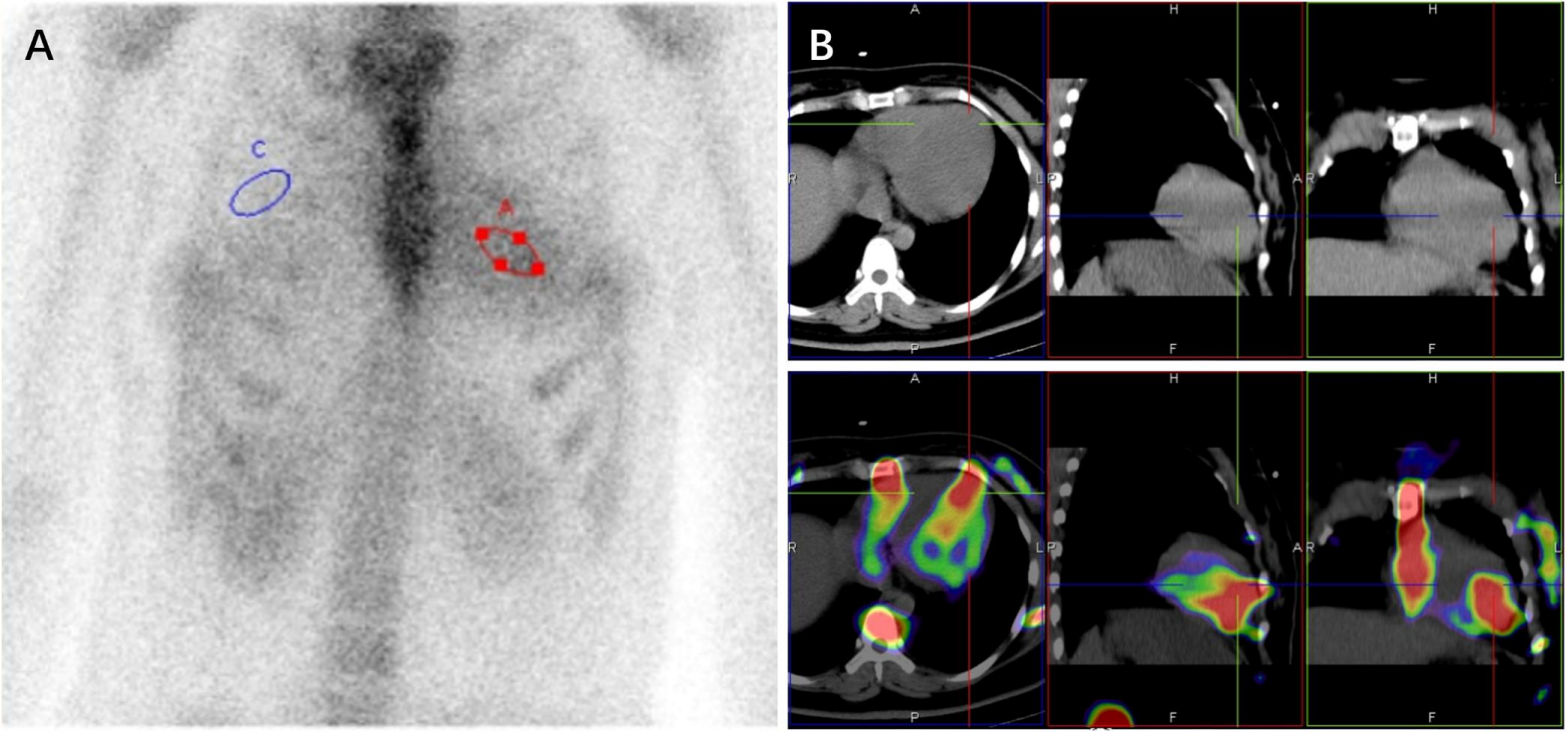
^99m^Tc-PYP scintigraphy **(A)** and SPECT/CT images **(B)** revealed a grade 3 myocardial uptake with heart (red circle) to contralateral lung (blue circle) ratio of 1.82 at 3hr **(A).**

Given the subendocardial LGE pattern on CMR, which raised suspicion for infiltrative disease including cardiac amyloidosis, Technetium-99 m pyrophosphate (^99m^Tc-PYP) scintigraphy was performed, which result demonstrated intense myocardial uptake [visual score Grade 3; heart-to-contralateral lung (H/CL) ratio: 1.82] (Figure 2). With immunofixation electrophoresis (IFE) of serum and urine revealing no presence of immunoglobulin monoclonal proteins, the positive result of ^99m^Tc-PYP scintigraphy seemed to indicate a potential diagnosis of transthyretin amyloid cardiomyopathy (ATTR-CM). Genetic testing detected no pathogenic variant in the *TTR* gene, effectively excluding hereditary ATTR (ATTRv-CM) and supporting wild-type ATTR (ATTRwt-CM). However, ATTRwt-CM typically affects older individuals, and the clinical presentation was atypical for several reasons: the patient was a young woman of reproductive age, and her EKG lacked low voltage. Furthermore, multiple reports describe false-positive results of ^99m^Tc-PYP scintigraphy in patients with hypertrophic cardiomyopathy (HCM) phenotype (5–11). These discrepancies prompted reconsideration of the diagnosis and led to the recommendation for further genetic testing to evaluate alternative etiologies.

**Figure 3:**
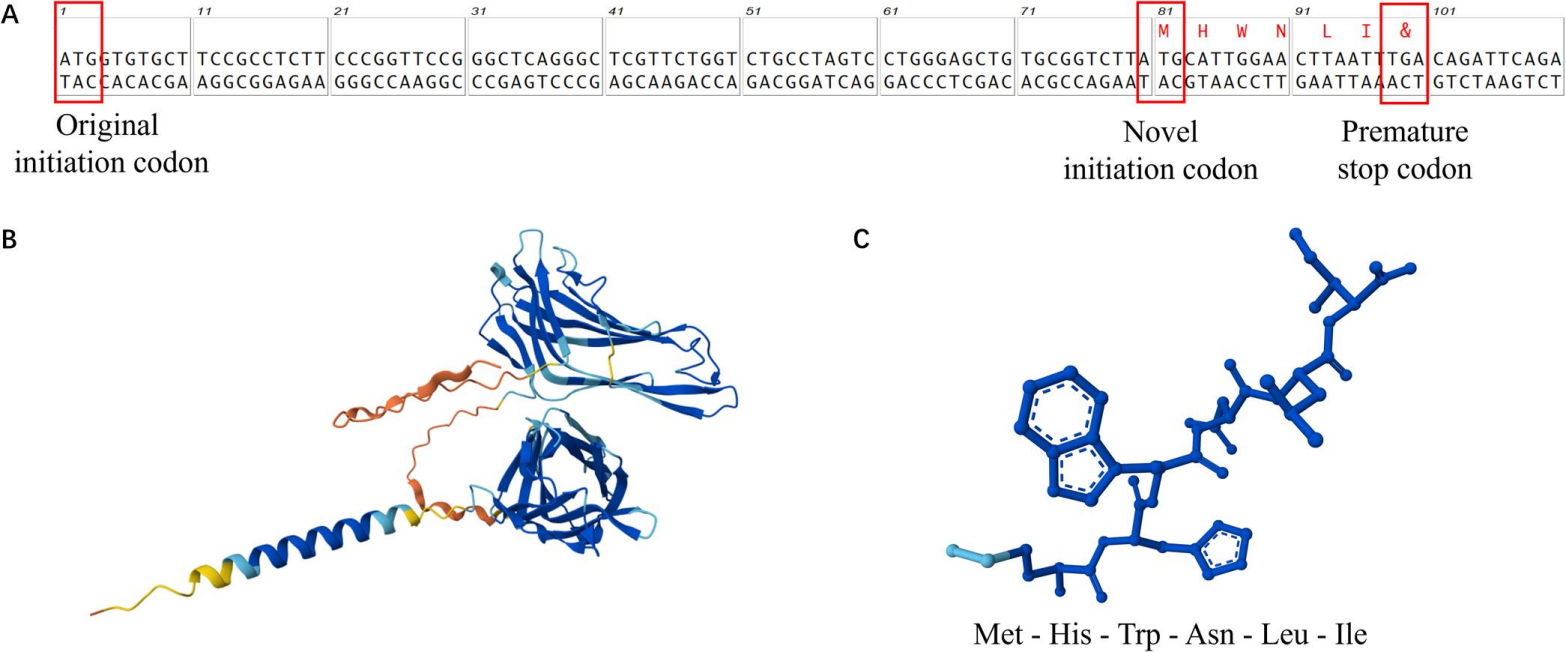
Schematic diagram of the new start codon **(A)** and the predicted structure of the LAMP-2 protein **(B)** compared with the mutant LAMP-2 protein **(C).**

Ultimately, whole-exome sequencing (WES) identified that the patient carries a heterozygous variant with a novel mutation in the lysosome-associated membrane protein-2 (*LAMP2*) gene: an A-T transition (c.1A > T) at the initiation codon (ATG) in exon 1. This variant was classified as suspected pathogenic (PSV1 + PM2) according to ACMG guidelines (12). We utilized MutationTaster to predict the mutation's functional impact on the LAMP-2 protein. As mutations in the start codon typically preclude translation initiation at the native site, a downstream ATG at positions c.80–82 was predicted to become the new translation start site. This shift results in a frameshift mutation, generating a truncated protein of only 6 amino acids before encountering a premature stop codon. Structure prediction by AlphaFold shows that the mutant protein lacks nearly all functional domains and is unlikely to retain its original function (Figure 3). The combination of this genetic mutation with the patient's phenotype of ventricular hypertrophy and conduction abnormalities strongly suggested the diagnosis of Danon Disease.

The patient was administered with oral spironolactone (20 mg once daily), Sacubitril/Valsartan (50 mg twice daily), Metoprolol succinate (23.75 mg once daily), Dapagliflozin (10 mg once daily), and Amiodarone (400 mg once daily) for arrhythmia management. At the 2-year follow-up, echocardiography demonstrated significant improvement, with the left ventricular ejection fraction (LVEF) reaching 65%. The patient remained free from heart failure-related hospitalizations and adopted a child from a local welfare institution.

## Discussion

Danon Disease is a rare, X-linked dominant inherited myopathy caused by defects in the *LAMP2* gene, which encodes lysosomal-associated membrane protein-2. Severe deficiency of this protein leads to cellular autophagy, accumulation of autophagosomes, mitochondrial dysfunction, and myocyte death (1).

To date, 472 distinct molecular consequences associated with *LAMP2* gene variants have been documented in the ClinVar Database, including 50 frameshifts, 248 missense, 38 nonsense, 22 splice sites and 114 untranslated regions. The majority of these mutations are pathogenic, with many definitively linked to Danon Disease. In this case, we identified a novel initiation codon mutation (c.1A > T) in *LAMP2* gene by whole-exome sequencing (WES). Our findings strongly suggested its pathogenicity for Danon Disease. This mutation, located within the initiation codon of exon 1, is classified as a missense initiation codon variant in ClinVar Database. Consistent with ACMG guidelines (12), it is categorized as suspected pathogenic (PSV1 + PM2). Notably, this specific mutation is absent from databases such as Exome Aggregation Consortium (EXAC) or Human Gene Mutation Database (HGMD).

In this case, this mutant *LAMP2* variant manifested solely as cardiac involvement without extracardiac symptoms. Cardiac conductive abnormality presented early, with a diagnosis of Wolff-Parkinson-White (WPW) Syndrome in her twenties. Hypertrophic cardiomyopathy and heart failure emerged only under physiological stress, specifically, during late pregnancy culminating in cesarean section. Apart from mildly elevated AST and LDH levels, no evidence of skeletal myopathy was found. The patient reported no visual impairment and displayed no cognitive deficits. However, phenotypic expression of this mutation exhibits marked gender disparity. Her deceased son displayed signs of developmental delay and cardiac dilation at one month of age. According to next-generation sequencing (NGS) at the local hospital, the boy was also a carrier of this *LAMP2* mutation and the clinical presentation strongly suggested he was succumbed to Danon Disease-related cardiomyopathy. Her mother's death around age 30 from suspected hypertrophic cardiomyopathy—likely attributable to this same *LAMP2* variant—further underscores the mutation's poor prognosis. Heterogenous penetrance and expressivity observed in female carriers may stem from the gene's X-linked dominant inheritance pattern, skewed X-chromosome inactivation and functional mosaicism in LAMP-2 expression (1, 2, 4, 13, 14). Another initiation codon mutation in *LAMP2* (c.2T > C) was recently reported by Wang et al. (15). Although both mutations (c.1A > T and c.2T > C) are predicted to disrupt the translation initiation codon, they are associated with strikingly divergent phenotypes. This observation suggests that additional factors may modulate phenotypic expression, warranting further mechanistic investigation.

False-positive results of ^99m^Tc-PYP scintigraphy are documented in multiple conditions (7–11, 16–21). The most common mimic is cardiac amyloidosis, typically light-chain (AL) type (6, 8, 17, 18), though rare etiology like apolipoprotein A-IV amyloidosis (19) and primary hyperoxaluria (causing myocardial oxalate deposition) (20) also occur. Other recognized causes include hyperphosphatemia (9), myocardial injury [e.g., infarction (21), direct current cardioversion (22)], and extra-cardiac tracer uptake from thoracic/pulmonary lesions (10, 11, 23, 24), leading to misjudgment of semiquantitative measurement. Notably, Danon Disease has recently emerged as a novel cause of PYP false positivity (7). Antukh D, et al. first reported this phenomenon in a 23-year-old man in 2020, who presented the classical triad of cardiomyopathy, polyneuropathy and myopathy, highly suggestive of glycogen storage disorder. In our case, however, the atypical cardiac symptoms and absence of extracardiac manifestations created significant diagnostic challenges, initially masking the systemic nature of her disease. This experience underscores that even highly specific tests like PYP scintigraphy can yield misleading results. Critically, this diagnostic pitfall reinforces the essential role of genetic testing in achieving precise etiological classification for undetermined cardiomyopathy.

The limitation is that we were unable to conduct a comprehensive assessment, such as ophthalmic images, electromyography and intelligence assessment. Endomyocardial biopsy was not clinically indicated. The patient has not returned for scheduled follow-up. Furthermore, additional studies should be conducted to investigate the underlying mechanism linking this variant to LAMP-2 protein dysfunction and clinical phenotype.

## Conclusion

In conclusion, this case study identified a novel initiation codon mutation in LAMP2 gene with strongly-suspected pathogenicity to Danon Disease. The first downstream ATG codon likely serves as an alternative translation start site, generating a truncated hexapeptide predicted to lack functional capacity in autophagy.

## Data Availability

The original contributions presented in the study are included in the article/Supplementary Material, further inquiries can be directed to the corresponding authors.

## References

[1] Hong KN Eshraghian EA Arad M Argirò A Brambatti M Bui Q International consensus on differential diagnosis and management of patients with danon disease. J Am Coll Cardiol. (2023) 82(16):1628–47. 10.1016/j.jacc.2023.08.014 37821174

[2] Endo Y Furuta A Nishino I. Danon disease: a phenotypic expression of LAMP-2 deficiency. Acta Neuropathol. (2015) 129(3):391–8. 10.1007/s00401-015-1385-4 25589223

[3] Huynh KK Eskelinen EL Scott CC Malevanets A Saftig P Grinstein S. LAMP Proteins are required for fusion of lysosomes with phagosomes. EMBO J. (2007) 26(2):313–24. 10.1038/sj.emboj.7601511 17245426 PMC1783450

[4] Brambatti M Caspi O Maolo A Koshi E Greenberg B Taylor MRG Danon disease: gender differences in presentation and outcomes. Int J Cardiol. (2019) 286:92–8. 10.1016/j.ijcard.2019.01.020 30857840

[5] Hanna M Ruberg FL Maurer MS Dispenzieri A Dorbala S Falk RH Cardiac scintigraphy with technetium-99m-labeled bone-seeking tracers for suspected amyloidosis. J Am Coll Cardiol. (2020) 75(22):2851–62. 10.1016/j.jacc.2020.04.022 32498813

[6] Poterucha TJ Elias P Bokhari S Einstein AJ DeLuca A Kinkhabwala M Diagnosing transthyretin cardiac amyloidosis by technetium Tc 99 m pyrophosphate. JACC: Cardiovascular Imaging. (2021) 14(6):1221–31. 10.1016/j.jcmg.2020.08.027 33221204 PMC8113330

[7] Antukh D Shchekochikhin D Rosina T Mershina E Larina O Pasha S Scintigraphy false-positive results for cardiac amyloidosis in a patient with danon disease. Clin Case Rep. (2021) 9(8):e04652. 10.1002/ccr3.4652 34430015 PMC8365861

[8] Zeng Y Poterucha TJ Einstein AJ Zhang Q Chen Y Xie H False positive technetium-99 m pyrophosphate scintigraphy in a patient with cardiac amyloidosis light chain: case report. Case Report. Medicine. (2021) 100(17):e25582. 10.1097/MD.0000000000025582 33907108 PMC8084032

[9] Hu LH Kuo Y Chang FP Wang WT Yang BH Huang WS Hyperphosphatemia-Related false-positive 99mTc-pyrophosphate myocardial scan: a case report with endomyocardial biopsy result. Clin Nucl Med. (2023) 48(11):e544–6. 10.1097/RLU.0000000000004869 37801577

[10] Nunes ARP Alves VM. Mitral annular calcification as a potential false-positive for cardiac amyloidosis in 99mTc-DPD scintigraphy accurately identified by SPECT/CT. Clin Nucl Med. (2024) 49(4):e179–81. 10.1097/RLU.0000000000005086 38350093

[11] Harford W Weinberg MN Buja LM Parkey RW Bonte FJ Willerson JT. Positive^99m^ tc-stannous pyrophosphate myocardial image in a patient with carcinoma of the lung. Radiology. (1977) 122(3):747–8. 10.1148/122.3.747 841065

[12] Richards S Aziz N Bale S Bick D Das S Gastier-Foster J Standards and guidelines for the interpretation of sequence variants: a joint consensus recommendation of the American college of medical genetics and genomics and the association for molecular pathology. Genet Med. (2015) 17(5):405–24. 10.1038/gim.2015.30 25741868 PMC4544753

[13] Cenacchi G Papa V Pegoraro V Marozzo R Fanin M Angelini C. Review: danon disease: review of natural history and recent advances. Neuropathology Appl Neurobio. (2020) 46(4):303–22. 10.1111/nan.12587 31698507

[14] D'souza RS Levandowski C Slavov D Graw SL Allen LA Adler E Danon disease: clinical features, evaluation, and management. Circulation Heart Failure. (2014) 7(5):843–9. 10.1161/CIRCHEARTFAILURE.114.001105 25228319 PMC4169002

[15] Wang Y Bai M Zhang P Peng Y Chen Z He Z Identification and functional analysis of a novel *de novo* missense mutation located in the initiation codon of *LAMP2* associated with early onset female Danon disease. Molec Gen & Gen Med. (2023) 11(9):e2216. 10.1002/mgg3.2216 PMC10496070 37288668

[16] Wu Z Xia S Ma D Li L. Negative methylene diphosphonate scintigraphy in biopsy-confirmed hereditary transthyretin Ala117Ser cardiac amyloidosis: a case report. Cardiology Plus. (2023) 8(3):206–10. 10.1097/CP9.0000000000000058

[17] Hsu TJ Tseng CT Kuo L Yang CY Lin YP Yu WC AHL Amyloidosis mimicking transthyretin amyloidosis on cardiac tc-99 m pyrophosphate scan: a diagnostic challenge. J Nucl Cardiol. (2025) 47:102147. 10.1016/j.nuclcard.2025.102147 39864580

[18] Arslan DF Inal E Isik EG Sanli Y. Extending amyloidosis diagnosis: a rare case of peritoneal AL amyloidosis revealed by 18F-FDG PET/CT and 99mTc-PYP SPECT/CT. Clin Nucl Med. (2025) 50(3):e192–3. 10.1097/RLU.0000000000005603 39639488

[19] Basel Allaw M Sinha A Ghafourian K Avery R Weinberg RL Lomasney JW Don’t judge a book by its cover: a case report of apolipoprotein A-IV cardiac amyloidosis. Eur Heart J Case Rep. (2023) 7(8):ytad341. 10.1093/ehjcr/ytad341 37681056 PMC10481775

[20] Wang YW Xie YT Sun XX. Hot hearts on bone scintigraphy are not all amyloidosis: hyperoxaluria-associated cardiomyopathy. Clin Nucl Med. (2024) 49(12):1120–1. 10.1097/RLU.0000000000005411 39192509

[21] Codini MA Turner DA Battle WE Hassan P Ali A Messer JV. Value and limitations of technetium-99 m stannous pyrophosphate in the detection of acute myocardial infarction. Am Heart J. (1979) 98(6):752–62. 10.1016/0002-8703(79)90474-5 495427

[22] Pugh BR Buja LM Parkey RW Poliner LR Stokely EM Bonte FJ Cardioversion and “false positive” technetium-99 m stannous pyrophosphate myocardial scintigrams. Circulation. (1976) 54(3):399–403. 10.1161/01.CIR.54.3.399 181172

[23] Zhou Q Zuo J Xiao L Jiang L Zhao Z. Bilateral multiple fractures obstruct quantitation of 99mTc-pyrophosphate imaging for cardiac amyloidosis. Clin Nucl Med. (2024) 49(7):e362–3. 10.1097/RLU.0000000000005230 38651782

[24] Khatri W Polydefkis M Vaishnav J Saad E Solnes LB Rowe SP. Large pleural effusion: a pitfall in the quantitation of 99mTc-PYP imaging for ATTR cardiac amyloidosis. Clin Nucl Med. (2022) 47(9):e594–5. 10.1097/RLU.0000000000004168 35384890

