# Supplementary material for: A novel LAMP2 initiation codon mutation causes Danon Disease: a case report
Source: Front Cardiovasc Med. 2025 Oct 8;12:1699732. doi: 10.3389/fcvm.2025.1699732 (PMC12540471; doi:10.3389/fcvm.2025.1699732)
Supplement: Supplementary file 1 [file Datasheet1.docx]

*Supplementary Material*

**Supplementary figures**


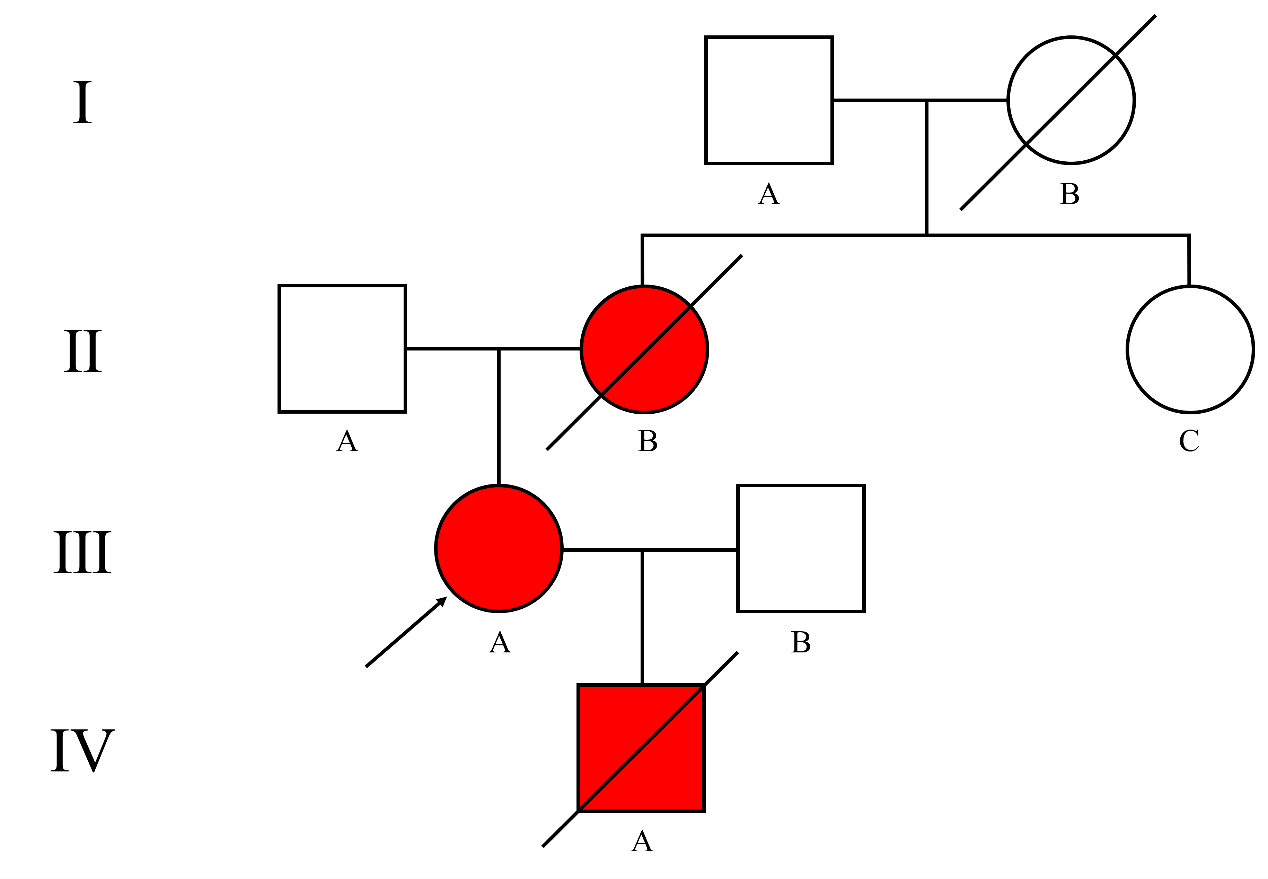


**Supplementary Figure 1.** Family pedigree of the patient shows an X-linked inherited pattern. Red-filled individuals are confirmed or suspected as cardiomyopathy patients. The patient (IIIA) is the proband of the family. IVA represents the patient’s prematurely deceased son due to cardiac issue. IIB represents the patient’s mother who died of suspectedly hypertrophic cardiomyopathy.


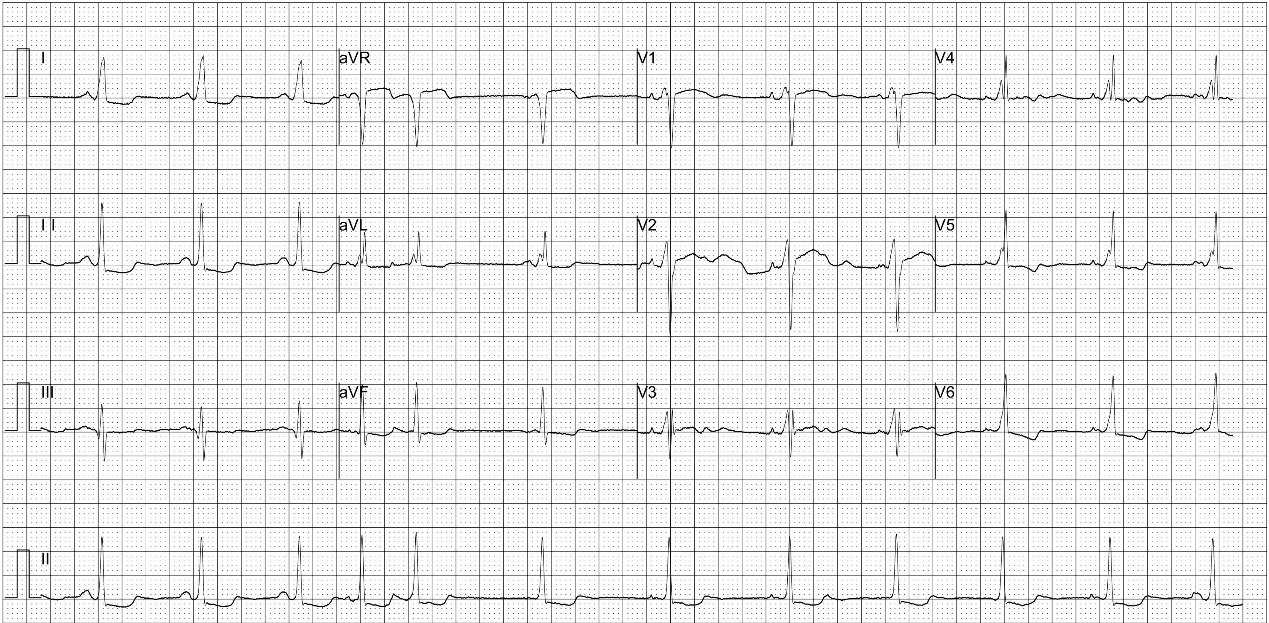


**Supplementary Figure 2.** Electrocardiogram showing atrial premature beat and ventricular pre-excitation.


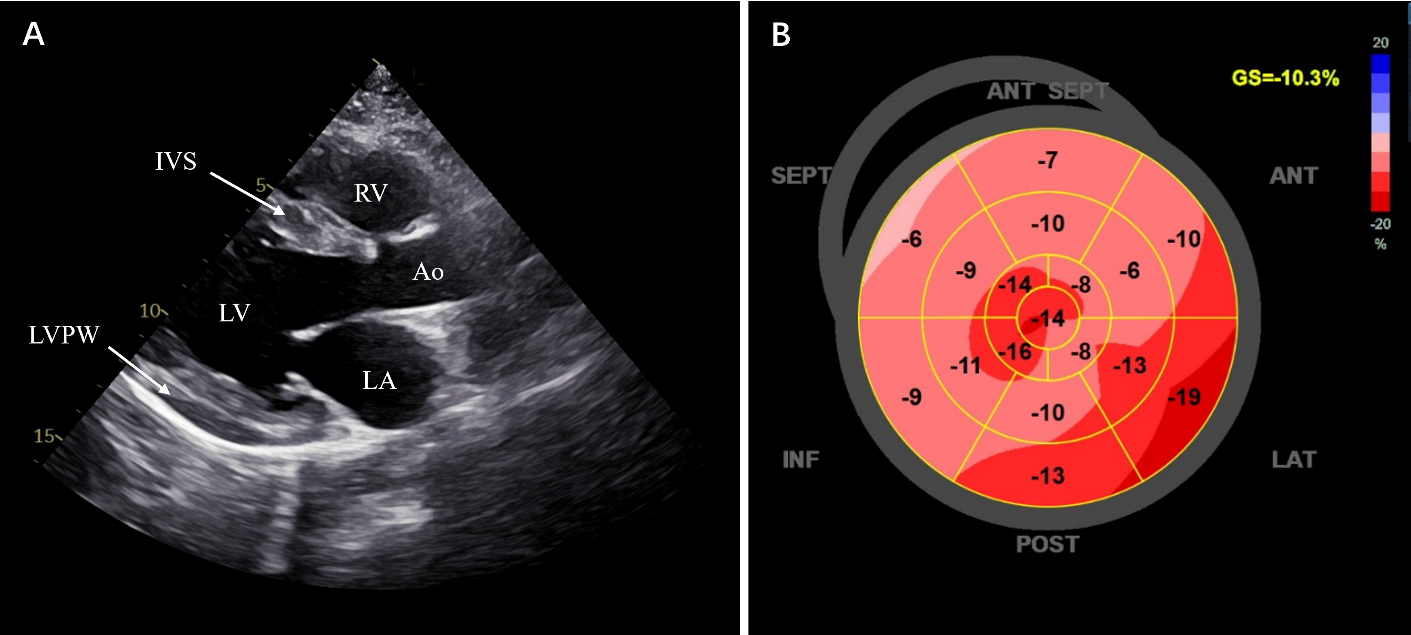


**Supplementary Figure 3.** Echocardiography showing mild left ventricular hypertrophy. Parasternal long-axis view(**A**) revealed thickness of 12mm in interventricular septum and posterior wall. Speckle-tracking echocardiographic image (**B**) showed a significantly lower longitude strain in the inferior wall and interventricular septum. Left ventricular global longitudinal strain (LV GLS) -10.3%. RV: Right ventricle; Ao: Ascending aorta; LV: Left ventricle; LA: Left atrium; IVS: Interventricular septum; LVPW: Left ventricular posterior wall; ANT SEPT: Anterior septum; SEPT: Septum; INF: Inferior wall; POST: Posterior wall; LAT: Lateral wall; ANT: Anterior wall;

**Supplementary table**

| **Component** | **Reference range** |  |
| --- | --- | --- |
| Leukocyte (×10^9^/L) | 3.69-9.16 | 7.30 |
| Erythrocyte (×10^12^/L) | 3.68-5.13 | 3.90 |
| Platelet (×10^9^/L) | 101-320 | 267 |
| Alanine Aminotransferase (IU/L) | 10-64 | 43 |
| Aspartate Aminotransferase (IU/L) | 8-40 | 45 |
| Blood Urea Nitrogen (mmol/L) | 2.5-7.1 | 3.9 |
| Creatinine (μmol/L) | 53-97 | 49 |
| Uric Acid (μmol/L) | 160-430 | 342 |
| Estimated Glomerular Filtration Rate (mL/min/1.73m^2^) |  | 125.9 |
| Sodium (mmol/L) | 130-147 | 137 |
| Potassium (mmol/L) | 3.50-5.10 | 4.12 |
| Chloride (mmol/L) | 96-108 | 102 |
| Lactate Dehydrogenase (IU/L) | 98-192 | 241 |
| Creatine Kinase Isoenzyme MB (ng/mL) | 0.3-4 | 3.3 |
| Myoglobin (ng/mL) | <70 | 16.1 |
| High Sensitivity-Troponin I (pg/mL) | <30 | 17.4 |
| N-terminal pro-B type Natriuretic Peptide (pg/mL) | 5-115 | 203.1 |
| C-reactive Protein (mg/L) | <5 | 3.61 |
| Urinary Albumin-to-Creatinine Ratio (mg/mmol) | 0-3.50 | 0.69 |
| International Normalized Ratio |  | 0.97 |
| D-Dimer (mg/L) | <0.55 | 0.28 |
| HbA1c (%) | 4.7-6.4 | 4.7 |
| Triiodothyronine (T3) (nmol/L) | 0.89-2.44 | 1.26 |
| Thyroxine (T4) (nmol/L) | 62.67-150.84 | 68.59 |
| Thyroid Stimulating Hormone (μIU/mL) | 0.3500-4.9400 | 2.2612 |
| Monoclonal Immunoglobulin (Blood and Urine) |  | negative |
| Free Light Chain (Blood and Urine) |  | negative |

**Supplementary Table 1.** Patient’s laboratory values at the time admitted.
